# Supplementary material for: High Prognostic Value of 68Ga-PSMA PET/CT in Renal Cell Carcinoma and Association with PSMA Expression Assessed by Immunohistochemistry
Source: Diagnostics (Basel). 2023 Sep 28;13(19):3082. doi: 10.3390/diagnostics13193082 (PMC10572927; doi:10.3390/diagnostics13193082)
Supplement: Supplementary file 1 [file diagnostics-13-03082-s001.zip › diagnostics-2565654-supplementary.pdf]

SUPPLEMENTARY MATERIAL

Table S1. Tumor characteristics at initial diagnosis.

No. T grade (%)	
1	5 (19.2)
2	5 (19.2)
3	15 (57.7)
4	1 (3.8)
No. T stage (%)	
1	5, T1a 4, T1b 1
2	5, T2a 4, T2b 1
3	15, T3a 13, T3b 2
4	1
No. pN category (%)	
N+	2 (7.7)
Nx	3 (11.5)
No. clinical M category (%)	
M0	4 (15.4)
M1 synchronic	5 (19.2)
M1 metachronic	16 (61.5)

Table S2. Site and uptake (SUVmax) of the target lesion in the enrolled patients with positive PSMA-PET.

**Patient**	**Site of the target lesion**	**SUVmax**
1	Liver (S2)	48.36
2	Left sacrum	46.37
3	Mediastinal node	35.44
4	Lumbar-aortic mass	66.6
5	Renal fossa	114.57
6	Sacrum	24.03
7	Ilium	10.2
8	Right adrenal gland	9.63
9	Renal fossa	7.4
10	Lung parenchima	7.4
11	Lung parenchima	8.17
12	Lung parenchima	24.94

13	Right kidney	9.96
14	Right kidney	54.25
15	Left kidney	5.45<br>5.45

Patient	Site of the target lesion	SUVmax
1	Liver (S2)	48.36
2	Left sacrum	46.37
3	Mediastinal node	35.44
4	Lumbar-aortic mass	66.6
5	Renal fossa	114.57
6	Sacrum	24.03
7	Ilium	10.2
8	Right adrenal gland	9.63
9	Renal fossa	7.4
10	Lung parenchima	7.4
11	Lung parenchima	8.17
12	Lung parenchima	24.94
13	Right kidney	9.96
14	Right kidney	54.25
15	Left kidney	5.45<br>5.45

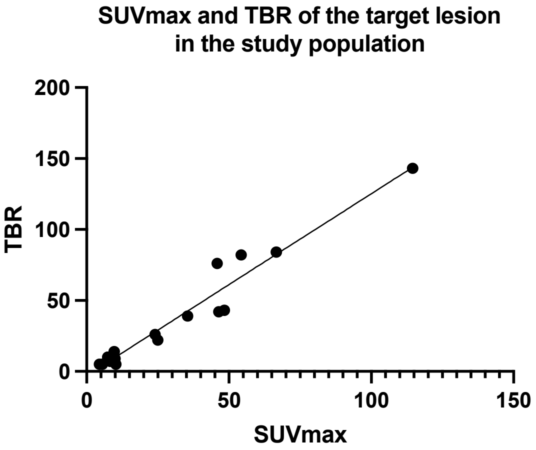

Figure S1. Behavior of SUVmax and TBR of the target lesion in subjects with positive PSMA-PET/CT
